# Organochlorine Compounds in the Amur (Heilong) River Basin (2000–2020): A Review

**DOI:** 10.3390/jox13030028

**Published:** 2023-08-20

**Authors:** Maksim M. Donets, Vasiliy Yu. Tsygankov

**Affiliations:** 1 School of Advanced Engineering Studies, Institute of Biotechnology, Bioengineering and Food Systems, Far Eastern Federal University, Ajax 10, Russky Island, Vladivostok 690922, Russia; donetc.mm@dvfu.ru; 2 Institute of the World Ocean (School), Far Eastern Federal University, Ajax 10, Russky Island, Vladivostok 690922, Russia

**Keywords:** POP, OCP, PCB, Amur, Heilong, Songhua, freshwater ecosystems

## Abstract

Persistent organic pollutants (POPs) are well-known contaminants that raise serious concerns, even more than 20 years after they were banned. Their worldwide distribution and persistence necessitate continuous monitoring in all components of the environment. The most challenging issues of POP regulation are associated with international water resources because their solutions require international cooperation in environment protection. This review provides data on various POPs (DDT, HCH, endrin, dieldrin, and PCBs) and their concentrations in aquatic organisms inhabiting the Amur River basin, one of the most poorly explored regions of Northeast Asia. Most studies have been conducted in the Songhua River (China), a tributary of the Amur River, which indicates that large inland bodies of water, especially those of international importance, require more extensive research.

## 1. Introduction

The Amur (Heilong) River is one of the most important rivers in Southeast Asia, and its basin is ranked among the world’s dozen largest river drainage basins. This body of water is of particular importance to the region due to its boundary location, great biodiversity, and the intensive use of the natural resources in its waters [1]. The latter is a substantial factor responsible for the current ecological condition of the Amur, causing a vast range of challenges in water management. The surface runoff from the catchment area in Russia and China makes the greatest contribution to the pollution in the river [2].

The ecological condition of the Amur River largely determines the quality and safety of biological resources harvested. As of 2019, 63.9% of the river monitoring stations were characterized as polluted and very polluted. In 2021, the situation substantially improved, and the level of combined pollution reduced to 50%, but the controlled parameters included a very limited list of pollutants. The state’s monitoring focuses mainly on inorganic pollution of the abiotic components of ecosystems [2]. In addition to the existing problems, an ecological disaster took place in the Amur River basin, and its consequences are still affecting the state of the river.

At the end of 2005, a series of explosions occurred at a petrochemical plant located at the Songhua River, Amur basin, in the city of Jilin, that produced benzene and its derivatives [3]. As a result, nitrobenzene, benzene, inorganic acids, and other compounds leaked into the Amur waters. To dilute the resulting mixture of toxic compounds, the Chinese authorities (together with the Russian side) made a decision to discharge water from the Chinese reservoirs. As a result, various pesticides, including those not used in Russia, got into the Amur River [4]. The Songhua River basin, where the explosions occurred, is still considered a supplier of toxic compounds to the Amur [3,5].

The People’s Republic of China is one of the major consumers and producers of pesticides worldwide [6]. Since the mid-20th century, organochlorine pesticides (OCPs) have been actively used there. The best-known representatives of this class of compounds are dichlorodiphenyltrichloroethane (DDT and its metabolites) and hexachlorocyclohexane (HCH), which were widely used in agriculture (to protect crops from pests) and medicine (to protect the population by controlling lice, malaria, and typhus vectors) [7,8]. Another well-known class of compounds is polychlorinated biphenyls (PCBs) that, due to their dielectric properties and persistence, were previously applied in a variety of industries in many countries worldwide, including Russia (the former USSR) and China [9]. Despite their high efficiency and almost ubiquitous use, OCPs and PCBs proved to be highly toxic to humans, as was evidenced by more in-depth toxicological studies. Subsequently, these compounds were included on the list of persistent organic pollutants (POPs) and banned for use in many countries across the world [10,11]. However, due to the lack of available and effective analogs, a number of developing countries, as well as China, have retained the right to use OCPs to control vectors of particularly dangerous diseases (such as malaria and typhus) [12].

Despite the exceptional importance of the Amur River from the aspects of fisheries management and supply of high-quality drinking water to the population, there have been very few POP studies on its basin. Our study aimed to summarize and analyze the data available in the literature on OCP and PCB levels in the Amur River basin for the period from 2000 to 2020 and to identify the possible sources of POPs in this water body.

## 2. Brief Characteristics of POPs

All POPs are highly volatile toxic xenobiotics distributed worldwide, which raises serious concerns. In recent decades, major attention has been paid to their distribution, sources, transformation, toxicity, and accumulation in terrestrial and aquatic ecosystems. Many of these chemicals are characterized by the potential to migrate with long-range atmospheric transport, in particular, from places where they are still applied [13]. By their chemical properties, POPs are inert substances (under normal conditions), resistant to concentrated acids and alkalis, and hydrophobic and stable in the environment [14].

POPs, as volatile substances, are studied mainly by gas chromatography methods. The typical methods for POP studies are gas chromatography with electron capture detector (GC-ECD) (most sensitive to halogenated compounds) [15,16], mass selective detector (including high-resolution mass detectors) (GC-MS, GC-MS/MS, GC-HRMS) [17,18,19], and time-of-flight detector (GC-TOF, GC Q-TOF) [20,21]. Also, due to their lyophilicity, all POPs dissolve in various solvents, which makes it possible to detect them using liquid chromatography with a mass selective detector (HPLC-MS/MS) (albeit on a much smaller scale compared to gas chromatography) [22,23].

Organochlorine pesticides (OCPs) are the most widely known POPs thanks to their pronounced insecticidal properties. The peak of their use was in the 1950s–1970s. These compounds can accumulate and be transferred up food chains (biomagnification) due to their chemical persistence and lipophilicity. According to estimates, eating contaminated food of animal origin (including aquatic organisms) accounts for more than 90% of OCP exposure in humans [11,24].

**Dichlorodiphenyltrichloroethane (DDT)** is probably the most notoriously famous organochlorine pesticide listed on the Stockholm Convention. It was applied not only in the agrotechnical sector but also in public health management, shipping, and other human economic activities [25].

After World War II, DDT was widely used in agriculture as an insecticide. Being the very first, well-known, and one of the most widespread pesticides, DDT caused large-scale contamination of water and soil resources, which led to serious deterioration of human health and affected ecosystems across the world [26]. In 2006, the World Health Organization (WHO) approved the use of DDT as a disease control agent in regions where malaria and other vector-borne pathogens pose a serious health threat. DDD and DDE are degradation products and metabolites of DDT. DDD was also produced and applied as an insecticide but to a much lesser extent than DDT. DDE, a product of DDT and DDD degradation, was not produced on an industrial scale. DDE is usually detected in the environment at concentrations often higher than the levels of the original compound [7].

**Figure 1:**
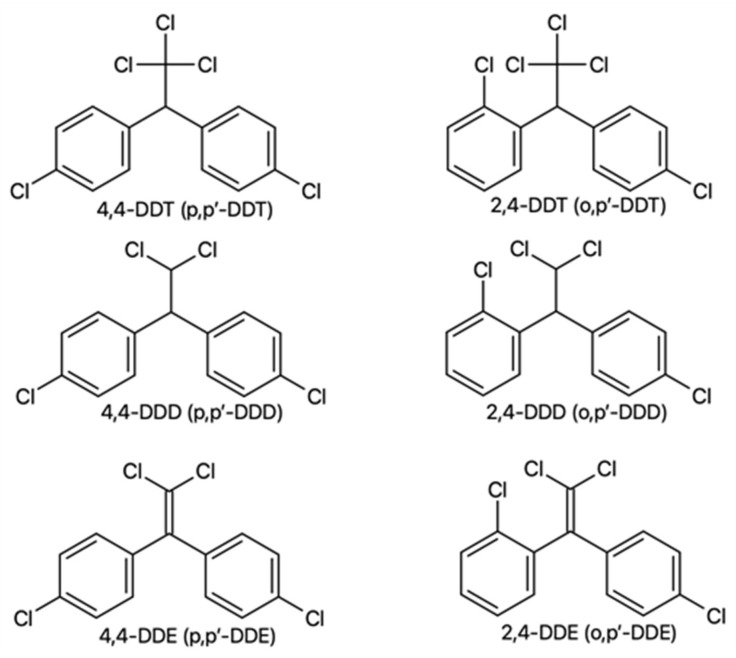
Chemical structures of DDT and its metabolites.

The main DDT formulation is a technical mixture of six isomers, of which the major portion consists of *p,p’*-DDT (77.1%) and *o,p’*-DDT (14.9%), differing in the position of chlorine atoms in benzene rings (Figure 1). The composition of a technical mixture includes, in addition to these two isomers, also *p,p’*-DDD (0.3%), *o,p’*-DDD (0.1%), *p,p’*-DDE (4.0%), and *o,p’*-DDE (0.1%) [7].

Despite the ban on the production and usage of DDT under the Stockholm Convention, some countries (in Asia, South America, and Africa) still retain the right to use DDT-based formulations for the control of disease vectors (malaria, typhoid, etc.) [12]. Therefore, there are constant discussions between scientists about the benefits of using DDT and its proven toxicity, including the chronic effects on human health [27,28]. The last decade can be characterized by the revision of the scientific opinion on DDT usage and its approval for further vector control in malaria-suffering countries, with the exclusion of its agricultural use [27].

**Figure 2:**
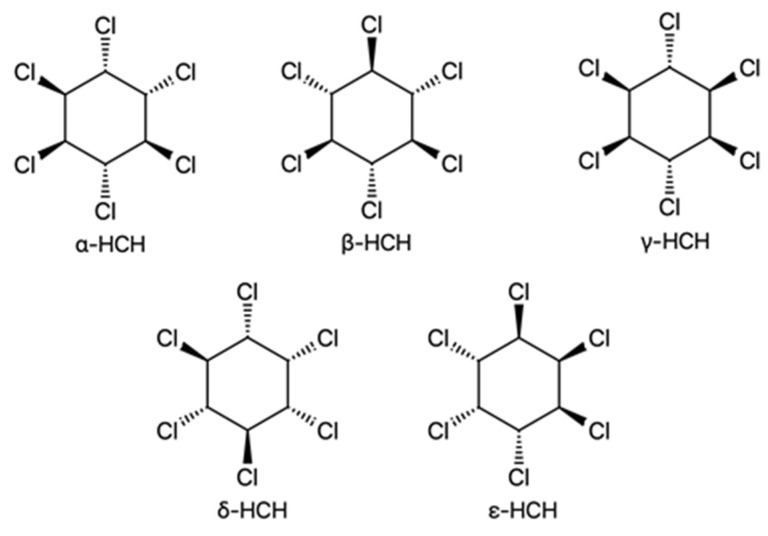
Chemical structure of HCH isomers.

Another pesticide that was widely and most actively applied in the period 1945–1990 is **hexachlorocyclohexane (HCH)** [29]. This compound was used both in the form of a technical mixture (containing a mixture of all isomers) and in a purified form in the lindane formulation (γ-HCH, after separation of isomers). The synthesized crude HCH contains a total of eight stereoisomers, from α- to θ-HCH, depending on the spatial arrangement of chlorine atoms (Figure 2). Among them, only α-, β-, γ-, δ-, and ε-HCH are stable; these are formed with the following percentages in reaction mixtures: α, 55–80%; β, 5–14%; γ, 8–15%; δ, 2–16%; and ε, 3–5%. The remaining three isomers are formed in trace amounts [30]. In the late 1940s and early 1950s, technical HCH mixtures were used as an insecticide. However, as was discovered shortly after, the use of such compositions resulted in the production of inedible cereals, vegetables, and fruits [31]. Of all the HCH forms, only γ-HCH exhibits pronounced insecticidal properties. Thus, beginning in the 1950s, some companies isolated it from a crude mixture and referred to the resulting compound as lindane [30]. In some countries, the transition to lindane began much later than the 1950s. For example, India used technical HCH until the late 1990s and then switched to the production and use of lindane. In China, the use of technical HCH was banned in 1983, and lindane has been applied since 1990 [32]. HCH is still produced and used in various formulations for the treatment of lice and scabies (mainly as a component of shampoos), which allows it to still enter the environment with household and industrial wastewater [8].

**Figure 3:**
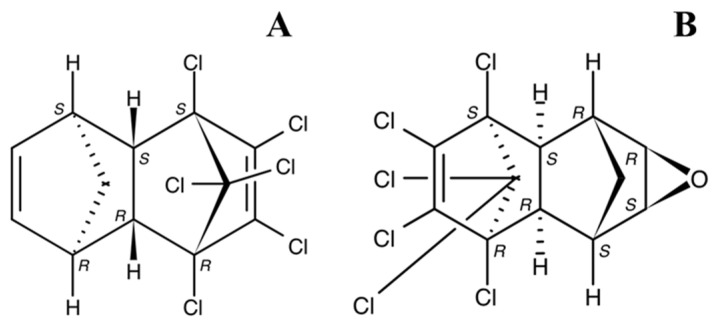
Chemical structures of aldrin (**A**) and dieldrin (**B**).

**Aldrin** and **dieldrin** are well-known organochlorine pesticides (Figure 3). These compounds are higher chlorinated insecticides applied to control various pests (moth larvae or termites) and to protect crops (corn, rye, barley, hay, etc.) [33]. Aldrin is unstable and easily turns into dieldrin (more than 56% of the initial concentration) under almost any conditions, as well as upon entering an organism, which makes it difficult to analyze in biological matrices [34]. Cyclodienes are moderately volatile and slowly biodegradable substances that have high soil adsorption coefficients and, as a result, exhibit low mobility in soils and a tendency to degrade in the water column. Aldrin and dieldrin are found in almost all environmental matrices [34]. Both compounds are capable of biomagnification. Residual amounts of these pesticides are detected in soil for a long time, as the concentrations of pollutants from dying organisms that have built up in them during their life cycles become gradually added to the already existing concentrations [35].

The scope of applications of these compounds is quite wide. For example, more than 50% of dieldrin produced in the USA in 1964 was used for pest control in the production of wool carpets and clothing, which contributed to long-term indoor air pollution [33]. These were also used as agents to protect agricultural products, as well as fruit and vegetable gardens, from pests.

According to some studies, dieldrin is one of the most persistent OCPs. Like DDT, dieldrin degrades in water to a limited extent, is almost not digested, and is not excreted from the body. Dieldrin is easily absorbed and circulates through the body with the blood or hemolymph of invertebrates [36].

**Figure 4:**
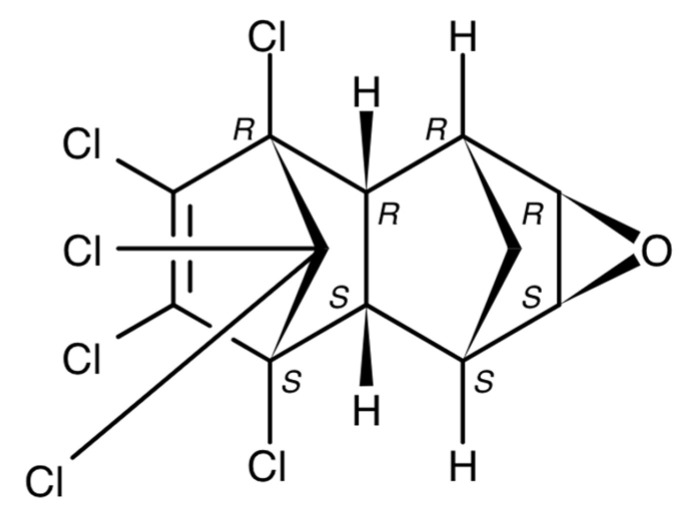
Chemical structure of endrin.

**Endrin** is a stereoisomer of dieldrin obtained through the interaction of hexachlorocyclopentadiene and vinyl chloride or acetylene [37] (Figure 4). This pesticide, which has rodenticidal, insecticidal, and biocidal properties, was actively applied to protect crops of sugar cane, tobacco, cotton, cereal, etc. [38]. Unlike aldrin and dieldrin, it was not used for termite control or other applications within urbanized areas. It is extremely toxic to birds and, therefore, was banned for use in 1991 [39].

Endrin is extremely persistent when entering soil [40]. It binds firmly to organic particles and is almost not leached under the effect of environmental factors. Nevertheless, it is capable of entering groundwater under certain conditions [39]. Approximately 20–30% of endrin evaporates immediately after being applied, but subsequently, its concentration does not vary.

Endrin exhibits pronounced biomagnification properties in the aquatic environment with, however, a relatively low biomagnification coefficient: its concentration increases twofold with each higher trophic level [41]. It also shows significant persistence and, nevertheless, can be degraded by microorganisms and algae with the formation of endrin ketone [42].

**Polychlorinated biphenyls (PCB)** are toxic xenobiotics that can currently be found in all regions on Earth. These compounds, as a rule, are heavy, high-boiling, oily liquids without odor and from transparent to yellowish in color. They are extremely inert and almost insoluble in water [43]. Due to their high thermal stability and good dielectric properties, PCBs were used for various industrial applications, in particular as lubricants, dielectric fluids, and plasticizers [9]. PCBs were widely produced for more than half a century, with a total PCB production around the world estimated at approximately 1.5 million tons. Due to their toxicity and persistence in the environment, these compounds were banned in most countries in the late 1970s [44].

**Figure 5:**
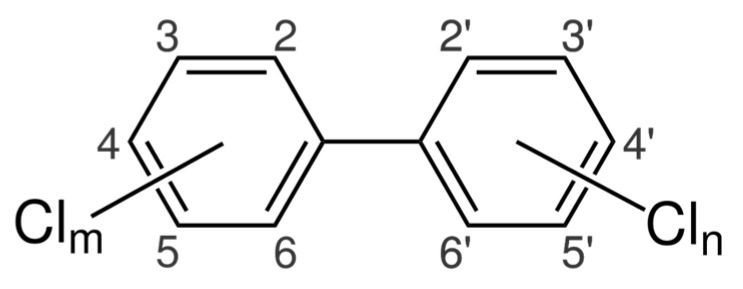
Chemical structure of PCBs.

PCBs comprise a group of homologues having a single structure (with two biphenyl rings) and differing only in the number and position of chlorine atoms (Figure 5). A total of 209 PCB homologues (also referred to as congeners) can be formed, containing from 1 to 10 chlorine atoms in various configurations [45], but only about 130 of them have been identified in environmental components to date [46].

Certain PCB congeners exhibit different physical and chemical properties, which eventually result in their different environmental behaviors and toxicities. The composition of technical mixtures depends on synthesis conditions and usually includes an extremely wide range of congeners. PCBs have a low solubility in water, which decreases with the increasing number of chlorine atoms in the molecule [47].

Two benzene rings in the biphenyl molecule can be oriented in the same plane or at an angle to each other (up to 90°). The number and location of substituents influence the degree of rotation of the benzene rings relative to the bond axis. Thus, the absence of chlorine atoms in *ortho*-positions or the presence of only a single substituent in the *ortho*-position (mono-*ortho*-substituted biphenyls) contributes to the retention of a flat structure, and such PCBs are referred to as planar or coplanar congeners [43]. As was suggested previously, the PCB toxicity rises with the increasing number of chlorine atoms in the molecule, but it has been proven that the stability, bioaccumulation potential, and toxicity of certain isomers depend on the position of chlorine atoms in the molecule. PCBs that lack chlorine atoms in *ortho*-positions, as well as mono-*ortho*-substituted PCBs (coplanar PCBs), have been found to be the most toxic of them [47].

Toxic substances can be formed as a result of PCB combustion: hydrogen chloride, dioxins, and dibenzofurans. It was found that pyrolysis of technical PCB mixtures led to the formation of some dibenzofurans. The latter are also byproducts of technical PCB synthesis [43].

Due to their high volatility and chemical stability, PCBs have become widely distributed by atmospheric transport. The circulation of PCBs in the environment has led to their presence and detection in almost all ecosystem components including air, water, soil, sediments, and live organisms. Due to their bioaccumulation in tissues and documented toxicity, PCBs are considered one of the major classes of environmental pollutants [9].

## 3. Sources of POPs in the Amur River Basin

The Amur, with its length of 4440 km and catchment area of 1,855,000 km^2^, is one of the largest rivers in Northeast Asia. It has an extremely extensive network of tributaries, of which the most important are the Argun, the Zeya, the Bureya, the Songhua, and the Ussuri (Wusuli) [48]. The river basin is located in the territories of three states: Mongolia, Russia, and China. The main users of its water operate on the Russian and Chinese sides [48,49]. These countries also make the major contributions to the pollution of this body of water. For example, according to the Amur Basin Water Agency, the river on the Russian side is exposed to pollutants from industrial enterprises located in large settlements such as, primarily, oil refineries, machine-building enterprises, and agricultural farms [50,51]. On the Chinese side, a significant pressure exists, in turn, in the middle reaches of the Amur River (the Songhua River estuary), where pulp-and-cardboard, petrochemical, and chemical plants producing chemical fertilizers, plastics, synthetic rubber, etc., are operated. Furthermore, intensive urban construction is underway, and there are also vast agricultural areas in Heilongjiang Province [52,53].

In general, it was previously noted that China continuously and in almost unlimited amounts discharged untreated industrial effluents into the Songhua River (due to the increase in production capacities) in the period from 1996 to 2006 that posed a threat of global environment disaster [54]. The pollution of the river was particularly acute until 2007, when the ecosystem degradation began posing risks to human health. After that, a number of laws were adopted at the state level (in China), and a study of water quality was carried out. This led to a substantial improvement in the situation as regards thetreatment of household wastewater and general pollution of the water body [53].

Nevertheless, a number of reports have confirmed that the major (up to 90%) source of Amur pollution is still the Songhua River, as evidenced by several indicators [5,55]. The condition of benthic communities in the river is observed to deteriorate, the water quality to decrease, and the turbidity to increase (especially downstream of the Songhua River estuary) due to the surface runoff of soil particulate matter from farmlands in Heilongjiang Province, China.

Despite the water abundance and sufficient water supply of the river, there is a quite pronounced competition for water resources in the Amur basin, primarily for uncontaminated water. Even in the most extensive Russian part, the population experiences a shortage of uncontaminated water downstream of the confluence of the Songhua River (flowing mainly through China), where a series of explosions occurred at the largest petrochemical plant in Jilin City, Northeastern China, on 13 November 2005. As a result, about 100 t of benzene and other chemicals was dumped into the water of the Songhua, with the full composition of the resulting mixture still remaining unknown [3,48]. During the passage of the so-called *benzene spot*, HCH, DDT, and other compounds were detected in the water samples from the river. Even after almost 20 years, the consequences of the accident continue to affect the condition of the main channel of the river network. This is annually confirmed by a substantial number of challenges in water management that still persist in the Amur and its largest transboundary tributaries, the Ussuri/Wusuli and Songhua, which have a determinative effect on the entire water management system in the basin of this river [2,5].

However, it is not only existing sources that contribute to the pollution of the river but also *historical* ones that formed as a result of the use or unintentional entry of various POPs into the water or sediments of the river in the past. For example, data have been published on the application of OCPs in the Russian part of the Amur during the period from 1987 to 1990. Approximately 12.1 t of HCHs was used here (in terms of active component), which is equivalent to 0.007 kg/km^2^. As regards DDT, no official data on the use are currently available. However, the total concentrations of DDT and its metabolites detected were substantial, while the ratio of the metabolites indicated a predominance of *historical* contamination with an insignificant or low influx of *fresh* DDT (as of 1988–1996) [56]. This information is also confirmed by Chinese researchers who attempted to estimate the amount of technical HCH, lindane, and DDT formulations used in the Amur River basin in the period from 1951 to 2000 [57]. As is noted in the paper, despite the ban on the use of DDT and technical mixtures of HCH, lindane (γ-HCH) and DDT-containing dicofol have been actively produced and used since 1990, thus supplying *fresh* forms of toxicants to the environment. According to the authors, in the period from 1952 to 1984, total amounts of 75,700 t of HCH (2227 t/yr), 3200 t of DDT (94 t/yr), and 94 t of lindane (9.4 t/yr in the period 1991–2000) were used in the Amur River basin. It is also noted that all the substances were applied exclusively within the agricultural sector, and not to control vectors of dangerous diseases.

Thus, the Amur River is continuously exposed to a range of various pollutants, including POPs. The extensive development of various industries, including agriculture, in the river basin has become one the major sources of current and historical pollution of this transboundary body of water. For a better understanding of the problem, we performed an overview of all available studies considering the spread of POPs in the Amur River basin in the period from 2000 to 2020. To the best of our knowledge, there are very few publications on this region, and they focus mainly on the abiotic components of ecosystems.

## 4. OCP and PCB Concentrations in Components of Ecosystems of the Amur River Basin

### 4.1. The Amur and the Ussuri

Surface waters are a major recipient of toxicants in the case of pollution of any water body. These are exposed to industrial, domestic, and terrigenous effluents, atmospheric transport, agriculture, etc. Moreover, water is a supplier of pollutants to other environmental matrices (e.g., to bottom sediments or organisms such as fish) or to other bodies of water (if flowing) [58]. Besides posing a potential threat to ecosystems, surface water pollution also becomes a public health concern [59].

Persistent organic pollutants are highly lipophilic compounds that are poorly soluble in water. Therefore, they are found mainly in the surface layer, being sorbed on organic particles, and in bottom sediments [7,8,9,34,39]. Thus, their concentrations in water, with the absence of sources of fresh entry, should be minimal.

The Amur (Heilong or Heilongjiang) is the largest river in Northeast Asia. It flows through Mongolia, China, and Russia from its source (the confluence of the Shilka and Argun rivers) in a northeasterly direction to its estuary, emptying into the Tatar Strait, Sea of Okhotsk, and, thus, it drains the largest basin for this sea. The largest tributaries of the Amur are the Zeya (Russia), the Bureya (Russia), the Amgun (Russia), the Songhua (China), and the Ussuri/Wusuli (Russia/China). The upper and middle Amur reaches are shared by Russia and China. The lower Amur reaches are located entirely within Russia’s territory, from the estuary of the Ussuri (Wusuli) River to the Amur Liman [60].

The Ussuri River is a large right-bank tributary of the Amur. Its length is 897 km and its catchment area is 193,000 km^2^, of which 57,000 km^2^ is located in China. The largest tributaries are the Muling (577 km in length, with a catchment area of 18,500 km^2^), the Bolshaya Ussurka (220 km, 29,600 km^2^), the Naoli (596 km), the Bikin (560 km, 22,300 km^2^), and the Khor (453 km, 24,700 km^2^). These rivers are mainly fed by rain, snow (by 5–20%), and groundwater (by 10–20%). The maximum river discharge values are recorded in May and August and rarely in July or September, and the lowest values in February and March. Lake Khanka is located in the Ussuri River basin. It is the largest standing body of water in the Russian Far East (with a water area of 4070 km^2^ and a catchment area of 18,400 km^2^) [61,62].

In one of the earliest studies of POP concentrations in the Amur River waters, an attempt was made to estimate the flux of OCPs from the Amur to the Sea of Japan and Sea of Okhotsk [56]. The researchers found that the amount of α- and γ-HCH in 1988–1996 made up 5.9 and 4.5 t, respectively. As regards DDT, data on the use are not available, but the influx of this pesticide in the Sea of Okhotsk for the same period (1988–1996) amounted to 10.2 t for both DDT and DDE. At that time, the DDE/DDT ratio in the river was approximately 1, which indicated the effect of predominantly historical pollution with an insignificant or low influx of *fresh* DDT [56].

Within the framework of assessment of the carcinogenic and noncarcinogenic risk from the Amur River water for residents of the region, concentrations of various POPs and the associated hazard to human health were estimated (Table 1) [63]. Unfortunately, accurate data were provided only for lindane (γ-HCH), whose concentration was 2 ng/L. For the rest of the pollutants, the authors indicated that their levels did not exceed the permissible levels in Russia (10 ng/L for natural water and 2000 ng/L for drinking water) [64]. It was also noted that the detected POPs were not used in Russia and could probably be considered tracers of anthropogenic pressure from China [63].

**Figure 6:**
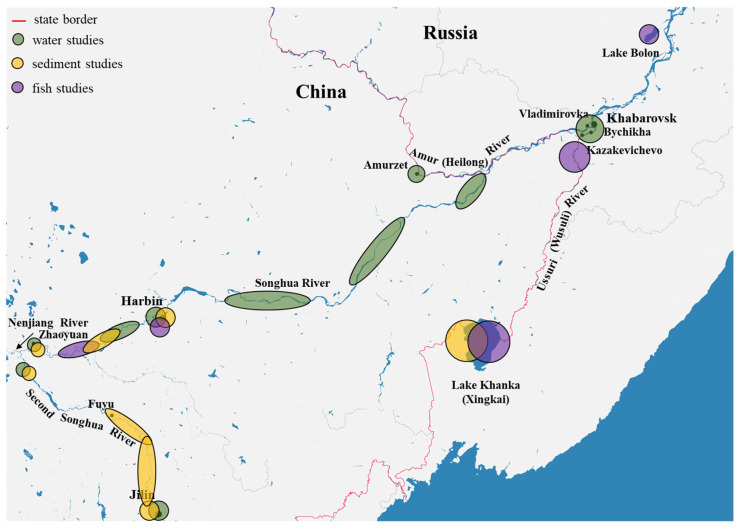
Sampling sites of known studies in the Amur River basin.

In 2011, the comprehensive Russian–Chinese monitoring of water quality of transboundary water bodies was carried out. The survey was conducted jointly by Russia and China in two catchment basins: the Amur River (Amurzet Village) and the Ussuri River (Kazakevichevo Village) (Figure 6). Among the 12 representatives of POPs studied, various OCP members, PCB congeners, etc., were found in the Amur water. In the Ussuri River, four POP representatives (not specified) were recorded. Despite a quite long list of compounds, only the maximum PCB concentrations are provided in the publication: 90 and 63 ng/L in the Ussuri and Amur rivers, respectively (Table 1) [65]. It is worth noting that both values exceed the permissible POP levels for natural water of fishery water bodies established in Russia (10 ng/L). It can also be seen that the PCB levels in the Ussuri River are higher than those in the Amur River, which indicates a substantial effect of the transboundary tributaries on the pollution of this water body.

The most recent data on pollutant levels in the waters of the Amur and Ussuri rivers are provided in the report by Mishchenko et al. (2022), who studied a wide range of POPs in these water bodies in 2019 and 2020 (Table 1) for several seasons [66]. In 2019, samples were taken in the summer and winter (Figure 6). Both rivers showed a decrease in DDT and aldrin concentrations (summer > winter) and an increase in HCH (winter > summer) between the seasons. The latter variation is probably explained by the continued entry of this pesticide directly into the water upstream and a decrease in its *evaporation* during the ice-cover formation. HCH is known to exhibit the highest volatility compared to other OCPs [8]. Thus, this pollutant can build up in the upper layer of water under ice, from where samples were collected. At that time, the levels of DDT and aldrin decreased (not significantly for DDT), which might be caused by their sedimentation with organic particles with the subsequent burial in bottom sediments. However, this theory can only be confirmed by a combined analysis of water and sediments in different seasons of the year.

In 2020, samples were taken in the summer, autumn, and winter seasons. Both the Amur and the Ussuri showed a decrease in DDT and aldrin concentrations in the following series: summer > autumn > winter. For HCH, the maximum levels were recorded in the autumn (by the end of the harvest season and during the season of heavy rains), which may be explained by the leaching of this pesticide from farmlands.

When comparing concentrations, one can clearly observe a significant increase in the levels of DDT (more than 20-fold), HCH (more than 10-fold), and aldrin (more than 2-fold) in the summer of 2020 (compared to 2019). It is worth noting that in 2019 and 2020, the POP concentrations did not meet the standards of the Russian Federation for natural waters (10 ng/L) and exceeded them 2- and approximately 50-fold, respectively [64]. The maximum levels of the toxicants in the summer–autumn period were most likely associated with flood events that occurred in the Amur River basin in 2019 and 2020. The water level at some observation points reached 8 m during flood events, which increased the terrigenous runoff (including that from agricultural areas). Furthermore, an increase in the Songhua River discharge and a release of water from the Bureya reservoir were documented, which could also affect the concentrations of pollutants [55].

Fishes from the Russian side of the Amur River basin have remained almost unstudied as regards POP content. Most studies were conducted in 2000–2010 and most did not provide scientific explanations for the concentrations reported or were fragmentary. For example, the DDT and HCH levels in sturgeon species from the Amur River were analyzed as part of a project aimed at creating a regulatory framework for food products from sturgeon of the Amur River basin (Table 2) [72]. It was found that the average content of pesticides did not exceed the permissible levels, but the study gave no scientific explanation for the concentrations presented, apparently due to the technological scope of the study. 

Data are available on the relationship between the consumption of polluted fish from the Amur River and the health status of the population in the period 1998–2004 [54]. The researchers analyzed more than 100 representatives of the commercial fish fauna from the river and more than 400 human samples and found a correlation between the rate of consumption and the prevalence of various pathologies in humans. As is noted in the publication, the levels of DDT and HCH were lower than the permissible ones in Russia (300 and 30 ng/g of wet weight (w.w.), respectively) and were detected in 20% of the fish samples analyzed, but the concentrations obtained were not provided. However, the conclusion itself about a probable potentially negative effect on public health necessitates continuous environmental monitoring of the fish resources in this water body. A similar study was carried out by Chukhlebova et al. (2005), who considered variations in values of hygienic parameters for commercial fish species [73]. In the period from 1998 to 2002, many of the fish caught from the Amur typically had a *chemical* odor that made it impossible to use them for food. The effect was especially pronounced in the winter season during the freeze-up period. Various hypotheses as regards the cause of the odor were considered. The concentrations of DDT and γ-HCH measured in fish were significantly lower than the permissible levels (300 and 30 ng/g w.w., Table 2). However, the authors suggested the immunosuppressive effect of a combination of chlorine-containing toxicants with the subsequent infection by pathogenic microflora (that was responsible for the *chemical* odor emitted by fish) [73,75]. This perfectly illustrates the negative influence of toxicants that exert a synergistic effect on natural ecosystems. The report also indicates the need to study the combined effects of low xenobiotic concentrations (below maximum permissible concentration (MPC) for food) on the fish body, the environmental risk they pose, and the risk to human health.

In 2006, the status of the fish fauna in Lake Bolon was assessed, which is a *transit* water body connecting delta lakes and rivers that empty into them with the main channel of the Amur River (Figure 6) [78]. Researchers selected four single fish samples (Table 2) and analyzed contents of certain toxicants in them. Of the POPs, only DDT was measured: its concentration in all the fish was 0.1 ng/g w.w. [75].

The most recent data on POP content in fish from the Ussuri River are provided by Zhang et al. (2016) [74]. In their study, the authors analyzed the levels of OCP and PCB in loaches (*Misgurnus mohoity* and *Paramisgurnus dabryanus*) inhabiting rice paddies at the Songhua and Ussuri rivers (Figure 6), where various OCPs (including DDT and HCH) were actively applied in the 1970s–1980s. The highest DDT and PCB concentrations were recorded from loach sampled in the Ussuri River, which may be explained by a higher bioaccumulation factor, as well as by the contamination of soil in rice paddies. The HCH levels in all loach were approximately similar between all the territories under study and did not depend on species (in the Songhua River). It is worth noting that, in all cases, the DDT concentrations were higher than the HCH concentrations, which differs from the patterns found in the more northerly areas (the Russian Far East and the adjacent seas) [79,80,81,82,83]. Furthermore, it is reported that the use of the aquatic organisms under study poses risks to the health of local farmers who actively consume loach as food.

Thus, in the Amur and Ussuri rivers, the POP concentrations in water increased significantly from 2010 to 2020. However, in 2020, the levels of toxicants varied between seasons tenfold, which makes it difficult to assess the dynamics of pollution of these rivers. We should also note here the actual lack of studies on bottom sediments of these rivers and an extremely small number of studies on fish fauna. The range of the studied matrices needs to be extended as well.

### 4.2. The Songhua

On the Chinese side, the major water body most frequently studied in the Amur River basin is the Songhua River. This river is located in Heilongjiang Province, Northeastern China. Due to its position in the northern, temperate monsoon zone, the river basin has a pronounced continental climate, characterized by a wide range of temperature variations throughout the year, with an average annual temperature within 3–5 °C, an average temperature in the rainy season of 20–25 °C, and an average annual precipitation of about 500 mm [53]. The Songhua is considered one of the most important sources of water for household use. Commercial fisheries, industrial production, and agriculture are actively developed here. Nevertheless, the Songhua is known as the major supplier of toxicants to the Amur River [55,76]. It is noted that in some areas, household wastewater is discharged directly into the river channel without proper treatment [71].

One of the first studies for the period under consideration that attempted to assess the spread of organochlorine pesticides on a national scale in China was carried out in 2003–2004 by Gao et al. (2008) [67]. The authors analyzed samples from more than 600 points in seven major river basins, including the Songhua River. Despite the rather negative evaluation of the surface water quality of this river, the authors noted a low rate of detection of lindane (in 17.5% of samples) and *p,p’*-DDT (in 5% of samples), without, however, providing a statistical processing of the results. According to their data, the approximate γ-HCH concentration in the Songhua water amounted to 71 ng/L, and the DDT concentration was lower than 0.14 ng/L. It is worth noting that the study was conducted before the accident at the Jilin Petrochemical Company that significantly affected the condition of the ecosystems in the Songhua and Amur rivers.

PCBs are among the most hazardous representatives of POPs. Despite their high toxicity, they are still often present in old electrical equipment (as a dielectric) and as additives in old dyes. These compounds can also be formed as a result of human industrial activity [84]. These toxicants are most readily selected for measurements in studies on the Songhua River ecosystems.

As part of the program for assessment of seasonal PCB distribution in the Songhua River, You et al. (2011) conducted a study of the lower reaches of this river from the boundary of Jilin Province to the border with Russia (Figure 6) [68]. Throughout the study area, the maximum PCB concentrations were recorded in the spring season (during ice melting, in April) and the minimum concentrations in the winter (Table 1). This may be explained by the determinative contribution of atmospheric transport and the local industrial facilities to the river pollution. In winter, when the water has no direct contact with the atmosphere, toxicants accumulate on the ice surface and then simultaneously enter the water as the ice melts. Subsequently, the pollutants that have entered the rivers are *washed away* by heavy atmospheric precipitation and the river flow, which reduces the PCB concentration almost twofold. Furthermore, a temperature increase can also reduce the level of pollutants through evaporation. However, the authors noted that the highest levels of toxicants were recorded downstream, from large industrial centers (the cities of Harbin, Yilan, and Jiamusi), and the minimum ones were upstream (Table 3). This indicates the direct contribution of industrial territories to the river pollution. It should also be noted that the concentrations in bottom sediments were significantly higher than in water. No seasonal variations in PCB concentrations between spring and summer were found, but differences in the qualitative composition of the detected pollutants were, nevertheless, recorded. In the spring, tetra- and pentacogeners made up the greatest portion of PCBs (in approximately equal proportions), while pentachlorinated biphenyls dominated in the summer. This was due to the lack of water/air interface during the freeze-up, the subsequent entry of toxicants into the water during ice melting, and their accumulation on bottom sediment particles.

In 2012, the DDT and HCH levels in the water of the main channel of the Songhua River (from the estuary of the Nenjiang River to Harbin, Figure 6) were 32 and 28 ng/g, respectively (Table 1) [69]. Thus, it can be stated that there was a significant increase in DDT concentrations and a decrease in HCH concentrations from 2003–2004 to 2012. However, the levels of the initial DDT in 2012 exceeded the concentrations of metabolites, which indicated a *fresh* source of the compound entering the environment. This could be caused by the use of antifouling DDT-containing paints [25,90,91] and also DDT-containing agents to control vectors of dangerous diseases and protect public health [69]. As regards HCH, both *historical* contamination and sources of fresh contamination were recorded. When considering bottom sediments, one can note an increase in DDT concentrations (from 1.1 to 2.4 ng/g of dry weight (d.w.)) and a significant decrease in HCH concentrations (from 5.0 to 2.0 ng/g d.w.), which indicates the above-described trends for water. It is likely that the Songhua River has a local source of DDT entering the environment, while HCH is gradually eliminated from the ecosystem through atmospheric or water transport [8,69].

The levels of *p,p’*-DDT and lindane (as well as some other OCPs) in the upper and lower reaches of the Songhua River were assessed as part of the development of the method for magnetic solid-phase extraction (magnetoliposome) of OCP from water [70]. Samples were taken during the seasons of rains (2014–2015) and drought (2015–2016) in the cities of Jilin and Harbin (the exact positions of the sampling sites were not provided) (Table 1). The levels of *p,p’*-DDT were below the detection limits in all the study areas. The concentration of lindane was 2.2 ng/L in the rainy season and 0.54 ng/L during the drought, which indicates the determinative effect of precipitation on the pesticide content of water. Since the coordinates of the sampling points were not specified in the study, we therefore provide an average concentration for the territory from Jilin to Harbin in our review. In general, the levels of pesticides are approximately similar to the concentrations recorded from the catchment area of the city of Khabarovsk (Amur River) in 2010–2011 (Table 1). In 2015, the same authors conducted a survey of levels of α-HCH (and other compounds) in the vicinity of Jilin and Harbin. However, as in the case of DDT, the toxicant was not detected in any of the samples studied [71].

Bottom sediments are considered a major reservoir of POPs in aquatic ecosystems. Due to their high affinity with organic particles, these compounds are adsorbed on them and settle to the bottom, forming a kind of *delay-action bomb* that can be activated at any time by stirring up the sediment [87,92]. Regular monitoring of concentrations in bottom sediments allows for the timely detection of variations and rapid identification of the source of pollution by tracking down the decrease in levels.

In 2005, a survey of bottom sediments was conducted in the upper (Jilin) and middle (Harbin) reaches of the Songhua River (Figure 6) [85]. The highest concentrations of all pollutants were recorded near the city of Jilin and downstream. Lindane was observed to dominate the HCH isomers in both the upper and middle reaches of the river (Table 3). DDE prevailed among DDT and its metabolites, which indicated a *historical* contamination. It should be noted that samples were taken shortly before the accident at the Jilin Petrochemical Company, which allows us to understand the condition of the ecosystem at that time. In general, all the locations sampled were characterized as seriously polluted. POPs typically showed a positive correlation with total organic carbon, which was also confirmed in the studies [84,87,93,94].

In a later study of the effect of a cement plant on the PCB content of bottom sediments in the Songhua River in the vicinity of Harbin, researchers reported extremely insignificant PCB concentrations (from 1.1 to 2.2 ng/g d.w.) with a quite wide range of congeners (35 various PCBs in total) [84]. In their more extensive study, they found the following trend of concentration variations from Jilin to Harbin: Fuyu > Jilin > Zhaoyuan > Harbin (Figure 6) [87]. The lowest PCB concentrations were recorded from bottom sediments sampled upstream of Jilin; then, the concentrations increased near the city area, and then increased fourfold near the city of Fuyu. Compared to the data of other authors, the PCB levels in the cities of Harbin and Jilin were two to four times as low [68,85]; in the city of Zhaoyuan, the values remained at about the same level and increased significantly in the area of the Second Songhua River near Fuyu (probably due to the existence of a local PCB source). This indicated an uneven human impact on the ecosystem of the Songhua River, an anthropogenic pressure on the river waters, and a decrease in the accumulation of toxicants in bottom sediments. It is also worth noting that the minimum PCB levels in the Second Songhua River remained unchanged during 1990–2005, after which there was a slight increase (from 1–2 to ~10 ng/g d.w.), probably associated with the accidents of November 2005 [95]. In the case of maximum concentrations, an increase has been observed since 1998 (from 1–2 to 123 ng/g d.w. in 2009), which is explained by the growing scale of activities of the industrial and chemical enterprises in the cities of Jilin and Fuyu [93]. However, in the main channel of the Songhua River, a steady decrease in concentrations in bottom sediments was observed for both minimum and maximum values [87]. It is worth noting that the initial levels of PCB pollution in the main channel were 20-fold higher than in the Second Songhua River, after which the level of pollution of the latter river grew annually. In general, a study of the mutagenic, carcinogenic, and genotoxic effects of water from the Songhua River showed all of them [58]. Even with the lack of exceedance of safety standards for the content of POPs (or other toxicants), these pollutants can show a cumulative effect, exerting mainly chronic toxicity and causing delayed consequences for ecosystems and human health [96,97,98].

Studies of fish from the Songhua River are very rare. Cai et al. (2014) measured DDT and HCH concentrations in the muscles of Amur carp caught from the main channel of the Songhua in 2012 [69]. The detected levels did not exceed the MPCs of Russia and China and amounted to 3.3 ng/g w.w. for DDT and 2.7 ng/g w.w. for HCH (Table 2) [80,82]. The higher DDT levels than the HCH ones proved to be consistent with the concentrations recorded from water and bottom sediment samples in the same study [69]. In a later study, Zhao et al. (2021) found that the levels of pollutants in Amur carp muscles significantly decreased to about 0.1 ng/g w.w. and varied slightly from year to year (Table 2) [76]. This might indicate a reduction in concentrations of POPs entering the Songhua waters due to the program for elimination of local sources of toxicants. Furthermore, the researchers detected endrin in all samples analyzed, which indicated its recent entry into the ecosystem and the possible presence of a local source of pollution.

Thus, the Songhua River was heavily polluted by various types of toxicants including aromatic compounds, polycyclic aromatic hydrocarbons (PAHs), and POPs, which were found in all segments of the river. After the Jilin Petrochemical Company accident in 2005, the Chinese government has made more efforts to address the environment management issues in the Songhua River basin. The number of treatment facilities has increased multifold, the discharge of untreated wastewater into the river has decreased, and industrial treatment facilities have been built to reduce amounts of pollutants. Currently, the water quality in the Songhua River is evidently improving.

### 4.3. Lake Khanka/Xingkai

One of the most important bodies of water in the Amur basin is Lake Khanka (Xingkai), connected to the river through the Songacha and Ussuri/Wusuli rivers. Lake Khanka is the largest freshwater lake in Northeast Asia. In 1976, Lake Khanka was included in the List of the Ramsar Convention on Wetlands of International Importance Especially as Waterfowl Habitat (1971). It is located in the southern part of the Sanjiang Plain in the Amur basin, at the Russia–China border, and is economically used by the two states. The area of the lake is 4070 km^2^. It is a shallow (on average, 2–3 m), loess-type body of water. A total of about two dozen rivers empty into the lake (e.g., the Muling, the Jinlyinku, the Xiaohei, the Ilistaya, and the Spassovka) and only one flows out, the Songacha, which connects the lake with the Amur River via the Ussuri River [77,99].

Khanka is a transboundary, Russian–Chinese basin-type lake (Figure 6). This explains a number of specifics and challenges in the functioning of the system and its condition, and also largely determines the environmental situation. The major areas of rice cultivation, both on the Russian and the Chinese sides, are located here. It is a region with the active development of various industries and a high concentration in the human population, especially in the Chinese part of the basin [100]. These factors have a substantial effect on the condition of landscapes, and also on coastal and aquatic ecosystems all over the Lake Khanka basin.

The Khanka basin remains almost unstudied as regards POP content. In 2011–2013, the OCP and PCB levels in bottom sediments of Lake Khanka amounted to 1.1 and 0.18 ng/g d.w., respectively, which are the lowest values for all the bodies of water under consideration (Table 3) [89]. It is worth noting that such a conclusion was rather expected, since the lake’s water is a protected area for both Russia and China. However, a study by Boyarova (2008) provides a number of DDT and HCH concentrations for various organisms inhabiting the Lake Khanka basin (in 2004) (Table 2) [77]. The highest levels of HCH, recorded from the liver of Amur pike, one of the main predators in the ecosystem, reached 722 ng/g w.w. The maximum HCH concentrations in the liver of yellowhead catfish amounted to 293 ng/g w.w. Among DDT and its metabolites, the maternal compound was detected quite frequently and accounted for up to 100% of ∑DDT recorded. In the case of HCH, most of the total concentrations (up to 80%) of the compound were also represented by the maternal compound (lindane), which indicates the existence of a local source of contamination in 2004. In addition to higher-order organisms, Boyarova also analyzed phyto- and zooplankton for OCP content and found only initial forms of DDT and HCH, which suggests the effect of atmospheric transport, as well as the influx of *fresh* toxicants from tributaries where rice paddies are still located [99]. Thus, despite the protected status, Lake Khanka has been exposed to significant anthropogenic pressure. Although the pollution exists, no analytical studies of POPs in the basin of this water body have been carried out to date.

## 5. Conclusions

Thus, the water bodies within the Amur River basin remain almost unstudied and are not described in the world’s literature as regards POP pollution. Despite the general improvement of the ecological condition in many tributaries, POP concentrations in the waters of the Amur/Heilong River (as well as in the Ussuri/Wusuli) have increased substantially. The probable relationship between the increase in concentrations of toxicants and flood events suggests the necessity to consolidate Russia’s and China’s efforts for addressing the issues of ecological safety of the transboundary water bodies and conducting joint scientific research on the identification and elimination of possible sources of pollution in order to protect the health of current and future generations.

## Figures and Tables

**Table 1:** Average concentrations of POPs in the water of the Amur River basin.

River	Sampling Site	Year	Study Period	Concentration, ng/L						Method	Detection Limits	References
				ΣDDT	ΣHCH	Aldrin	Dieldrin	Endrin	ΣPCB			
Amur/Heilong River	Khabarovsk, Khabarovsk Krai	2010	Not specified	<10 ^1^	2 ^2^	<10	<10	<10	<10	NA ^3^	NA	[63]
	Amurzet village, Jewish Autonomous Oblast	2011	Not specified	– ^4^	–	–	–	–	63 ^5^	NA	NA	[65]
	Vladimirovka village, Khabarovsk Krai	2019	Summer	20	13	40	–	–	–	GC-ECD	NA	[66]
			Winter	16	21	<10	–	–	–			
		2020	Summer	777	161	82	–	–	–			
			Autumn	279	24	28	–	–	–			
			Winter	73	18	–	–	–	–			
Ussuri/Wusuli River	Kazakevichevo village, Khabarovsk Krai	2011	Not specified	–	–	–	–	–	90 ^5^	NA	NA	[65]
	Bychikha village, Khabarovsk Krai	2019	Summer	36	15	73	–	–	–	GC-ECD	NA	[66]
			Winter	23	48	<10	–	–	–			
		2020	Summer	372	14	59	–	–	–			
			Autumn	342	23	21	–	–	–			
			Winter	99	19	<10	–	–	–			
Songhua River	Not specified	2003–2004	Not specified	<0.14	71 ^2^	–	–	–	–	GC-μECD	0.11–0.17 ng/L	[67]
	Mainstream of the Songhua River	2007	Spring	–	–	–	–	–	10	GC-MS	0.003–0.032 ng/g	[68]
			Summer	–	–	–	–	–	5.5			
		2008	Winter	–	–	–	–	–	4.4			
	Mainstream of the Songhua River (from the mouth of the Nenjiang River to Harbin)	2012	Summer	32	28	–	–	–	–	GC-ECD	NA	[69]
	Jilin, Harbin (not specified)	2014–2015	Rainy season (September, June, August)	<DL ^6^	2.2 ^2^	–	–	–	–	GC-MS/MS	0.04–0.37 ng/L	[70]
		2015–2016	Dry season (March, October, April)	<DL ^6^	0.5 ^2^	–	–	–	–			
	Harbin	2015	September–October	–	<DL ^7^	–	–	–	–	GC-MS/MS	0.1–0.15 ng/L	[71]
	Jilin			–	<DL ^7^	–	–	–	–			

^1^ According to [64]; ^2^ lindane only; ^3^ not available; ^4^ not studied; ^5^ maximum concentrations; ^6^ *p,p’*-DDT only; ^7^ α-HCH only; DL, detection limit.

**Table 2:** Average concentrations of POPs in fish from the Amur River basin.

River/Lake	Species	Organ	Year	Concentration, ng/g Wet Weight						Method	Detection Limits	References
				ΣDDT	ΣHCH	Aldrin	Dieldrin	Endrin	ΣPCB			
Amur/Heilong River	Sturgeon ^1^	Not specified	2000	20	10	– ^2^	–	–	–	NA ^3^	NA	[72]
	Redfin, catfish, barbel steed ^4^	Muscle	2002	0.062–0.075 ^5^	–	–	–	–	–	NA	NA	[73]
	Predatory carp, barbel steed			–	0.023–0.025 ^5^	–	–	–	–			
Ussuri/Wusuli River	Loach (*Misgurnus mohoity*)	Whole body	2016	14.7	1.4	0.0002	–	–	0.44	HRGC-HRMS	0.000023–0.11223 ng/g dry weight (dw)	[74]
Lake Bolon ^6^	Prussian carp (*Carassius gibelio*)	Muscle	2006	0.0001	–	–	–	–	–	NA	NA	[75]
	Amur pike (*Esox reichertii*)			0.0001	–	–	–	–	–			
	Barbel steed (*Hemibarbus labeo*)			0.0001	–	–	–	–	–			
	Roach (*Rutilus rutilus lacustris*)			0.0001	–	–	–	–	–			
Songhua River	Amur carp (*Cyprinus rubrofuscus*)	Muscle	2012	3.3 ^7^	2.7	–	–	–	–	GC-ECD	NA	[69]
	Loach (*Misgurnus mohoity*)	Whole body	2016	4.2	1.5	0.0002	–	–	0.33	HRGC-HRMS	0.000023–0.11223 ng/g dw	[74]
	Large-scale (*Paramisgurnus dabryanus)*			4.1	1.3	0.0002	–	–	0.29			
	Amur carp (*Cyprinus rubrofuscus*)	Muscle	2018	0.0001	0.11	–	–	0.079	–	NA	NA	[76]
			2019	0.06	0.10	–	–	0.034	–			
			2020	0.13	0.11	–	–	0.049	–			
Lake Khanka/Xingkai	Mussel (*Cristaria herculea*)	Soft tissues	2004	27	13	–	–	–	–	GC-ECD	NA	[77]
	Yellohead catfish (*Pseudobagrus fulvidraco*)	Liver		293	360	–	–	–	–			
	Predatory carp (*Chanodichthys erythropterus*)	Liver		216	130	–	–	–	–			
	Amur pike (*Esox reichertii*)	Liver		173	722	–	–	–	–			
	Spotted steed (*Hemibarbus maculatus*)	Liver		99	32	–	–	–	–			
	Phytoplankton	–		51 ^8^	281 ^9^	–	–	–	–			
	Zooplankton			30 ^8^	12 ^9^	–	–	–	–			

^1^ Species not specified; ^2^ not studied; ^3^ not available; ^4^ average concentration for all species; ^5^ maximum value range; ^6^ single samples; ^7^ total of *p,p’*-DDE, *p,p’*-DDD, *p,p’*-DDT, and *o,p’*-DDT; ^8^ DDT only; ^9^ lindane only.

**Table 3:** POPs concentrations in bottom sediments of the Amur River basin.

River/Lake	Sampling Site	Year	Study Period	Concentration, ng/g Dry Weight						Method	Detection Limits	References
				ΣDDT	ΣHCH	Aldrin	Dieldrin	Endrin	ΣPCB			
Songhua River	Second Songhua River (Jilin)	2005	Summer	2.1	6.2	0.26	1.6	1.2	7.5	GC-ECD GC-MS	0.01–0.8 ng/g	[85]
			Not specified	– ^1^	–	–	–	–	3.2–85.9 ^2^	NA ^3^	NA	[86,87]
	Mainstream of the Songhua River (Harbin)	2005	Summer	1.1	4.9	0.10	0.57	0.48	3.1	GC-ECD GC-MS	0.01–0.8 ng/g	[85]
		2006	Not specified	–	–	–	–	–	0.40–16.70 ^2^	NA	NA	[86,87]
		2008	Not specified	–	–	–	–	–	1.7–6.3 ^2^	NA	NA	[87,88]
	Mainstream of the Songhua River (from the mouth of the Nenjiang River to Harbin)	2007	Spring	–	–	–	–	–	6.1	GC-MS	0.003–0.032 ng/g dw	[68]
			Summer	–	–	–	–	–	5.03			
		2012	Summer	2.38	2.04	–	–	–	–	GC-ECD	NA	[69]
	Harbin	2014	Summer	–	–	–	–	–	1.6	GC-NICIMS	0.003–0.032 ng/g dw	[84]
	Harbin		Summer	–	–	–	–	–	2.3	GC-NICIMS	0.003–0.032 ng/g dw	[87]
	Zhaoyuan			–	–	–	–	–	3.4			
	Fuyu			–	–	–	–	–	12.4			
	Jilin			–	–	–	–	–	3.7			
Lake Khanka/Xingkai	Not specified	2011–2013	Not specified	0.8	<DL ^4^	0.3 ^4^			0.18	GC-MS triple quad	0.003–0.041 ng/g C^13^ isotope standard used	[89]

^1^ not studied; ^2^ concentration range; ^3^ not available; ^4^ total of aldrin, dieldrin, endrin, and other OCPs; DL, detection limit.

## Data Availability

Data sharing is not applicable to this article.
